# The prevalence of insomnia symptoms in adults with depression: a systematic review

**DOI:** 10.1192/j.eurpsy.2025.906

**Published:** 2025-08-26

**Authors:** M. Toynbee, D. Zygmunt, R. Levett, S. Moghaddacy, S. Pappa

**Affiliations:** 1 West London NHS Trust; 2 Imperial College London, London, United Kingdom

## Abstract

**Introduction:**

Insomnia symptoms are commonly reported by people with depression, and their effective treatment is associated with reduced affective symptoms and improved patient outcomes. However, to date, estimates of the rates of co-morbid insomnia in individuals with depression are mainly based on sub-analyses of epidemiological studies of general populations, often relying on self-reported or historical depression diagnoses. To our knowledge there has been no systematic review of studies evaluating the prevalence of insomnia symptoms in people with an established diagnosis of depression.

**Objectives:**

To summarise and synthesize the available evidence of studies reporting on the prevalence of insomnia symptoms in adults with depression using a systematic review.

**Methods:**

Comprehensive searches of MEDLINE, EMBASE, Pubmed, PsycINFO, and TRIP using search terms related to depression and insomnia with no language restrictions, were performed. The titles and abstracts were screened independently by two reviewers and the full texts of studies identified were assessed against predefined criteria, namely: 1) primary studies of 2) adults (18 years and over) with depression, 3) reporting prevalence of insomnia symptoms. The review protocol was registered with Prospero.

**Results:**

4,477 unique references were identified, of which 80 full texts were reviewed after title and abstract screening. Thirteen studies, with a combined total of 10,394 participants and published between 2001 and 2023, met the inclusion criteria and were included in the analysis. Seven studies were from the US, and one each from China, Turkey, S. Korea, Belgium, and the Netherlands, while one included participants from both Malaysia and Australia. The mean prevalence rate of clinically significant insomnia symptoms in adults with a diagnosis of depression was 79%. The systematic review has a number of limitations including a small number of eligible studies covering a wide range of geographic populations with varying thresholds for reporting insomnia, and that establishing the prevalence of insomnia symptoms was not the primary aim of most of the studies. Further meta-analysis in this research was not carried out due to high heterogeneity between studies.

**Conclusions:**

To our knowledge, this is the first systematic review of studies reporting on the prevalence of insomnia symptoms in adults with depression. The main finding is that at least three in four adults with depression have clinically meaningful insomnia symptoms; such high prevalence rates are consistent with previously reported figures. Therefore, given the positive clinical outcomes from treatment, services for adults with depression should routinely offer targeted interventions in identifying and managing co-incident insomnia symptoms. Future research should aim to refine the estimates of co-occurrence of insomnia and depression in different clinical populations.

**Disclosure of Interest:**

None Declared

